# Dr. Satish S. Maniyar

**Published:** 2010

**Authors:** S S C Chakra Rao

**Affiliations:** Hon. Secretary, ISA

**Figure:**
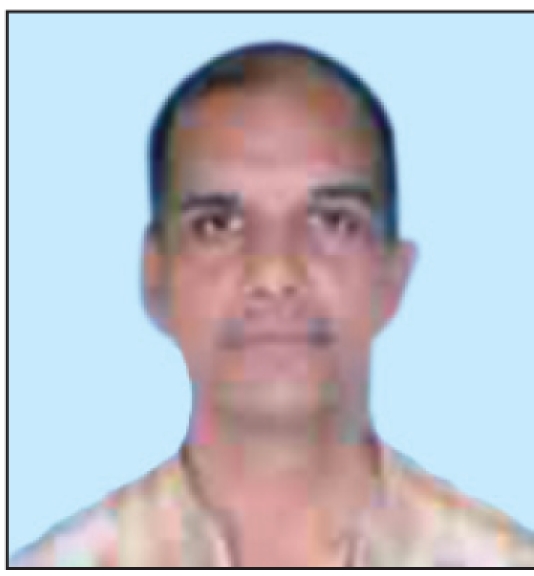


With a deep sense of regret, we inform the sad demise of Dr. Satish S. Maniyar on 22^nd^ July 2010. Born on 26^th^ November 1958, Dr. Satish S. Maniyar was an active life member (M1837) of Indian Society of Anaesthesiology, Parbhani, Maharashtra.

May his soul rest in peace.

